# Risk assessment of norovirus and hepatitis A virus in strawberries imported into China

**DOI:** 10.1002/fsn3.3721

**Published:** 2023-10-07

**Authors:** Junjie Zhong, Yunfeng Yang, Hui Zhang, Shuwen Zhang, Xiaosheng Qu, Qin Chen, Bing Niu

**Affiliations:** ^1^ School of Life Sciences Shanghai University Shanghai China; ^2^ National Engineering Laboratory of Southwest Endangered Medicinal Resources Development Guangxi Botanical Garden of Medicinal Nanning China

**Keywords:** critical control point system, QMRA, strawberries, supply chain, virus

## Abstract

Norovirus (NoV) and hepatitis A virus (HAV) pose a considerable health risk worldwide. In recent years, many cases of virus infection caused by virus‐contaminated strawberries have occurred worldwide. This study applied a critical control point system to analyze the main hazards during the production and marketing of strawberries imported into China and explore the key control points in the whole process. To further evaluate the risks in the supply chain, the established quantitative microbial risk assessment (QMRA) was used to determine the probability that residents would be infected with viruses after consuming imported strawberries. It was found that the risk of virus contamination from imported strawberries was low, and the virus contamination mainly results from water resources and personnel. This research helps the regulatory authorities formulate strategies to ensure the long‐term microbial safety of imported strawberries. In addition, the methods may prove useful in evaluating the risks of other agricultural produce.

## Graphical abstract

Enteroviruses are the main cause of outbreaks of foodborne diseases. We investigated the risk of norovirus and hepatitis A virus in imported strawberries based on the supply chain from both qualitative and quantitative perspectives. In the strawberry supply chain, the virus contamination factors were determined by the critical control point system.

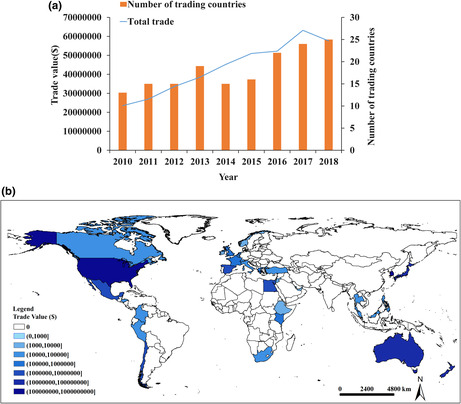

## 1 INTRODUCTION

**FIGURE 1:**
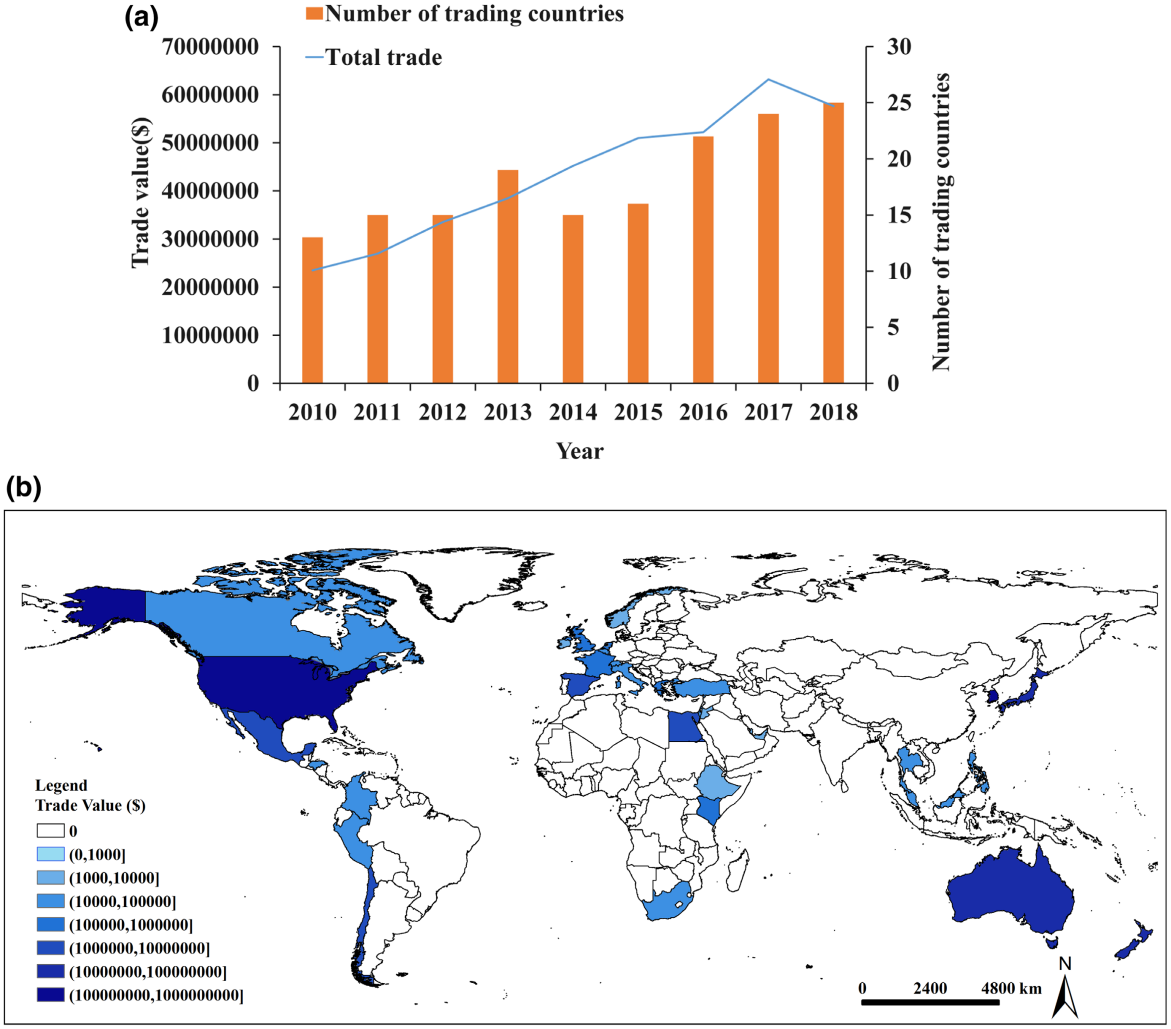
Trade status of imported strawberries in China from 2010 to 2018. (a) Source countries and total trade value of imported strawberries, (b) total trade value of strawberry exporting countries from 2010 to 2018.

Norovirus (NoV) and hepatitis A virus (HAV) have been identified as enteric viruses associated with most outbreaks of foodborne diseases (Li et al., 2018). NoV is the main cause of acute gastroenteritis worldwide, and it is related to about one fifth of cases in occident are caused by NoV (Bosch et al., 2018). HAV is the cause of viral hepatitis, which causes vomiting and diarrhea (Kulsuptrakul et al., 2021). With the increasing trade of strawberries between China and other countries (https://oec.world/; Figure 1a), the food safety of strawberries has become increasingly important.

Many factors, including food handlers, water resources, and the environment, polluted strawberry supply chain (Bartsch et al., 2018). At present, the major problem is tracking strawberry information in real time and predicting the outbreak of foodborne diseases (Wang et al., 2013). Hence, it is helpful to develop a safety risk assessment model of strawberries to assess the risk of virus contamination before the outbreak of a disease.

The critical control point system is a preventive food safety control method to identify and evaluate food production and processing hazards. However, critical control point system cannot quantitatively analyze the virus contamination risk of strawberries. Quantitative microbial risk assessment (QMRA) is a systematic method for assessing public health risks caused by exposure to microbial pathogens. The QMRA allows uncertain factors to be incorporated and propagated into the model using input values following a certain frequency or probability distribution function and the Monte Carlo simulation (de Matos Nascimento et al., 2020).

Here, the critical control point system combined with QMRA based on factors of environmental virus contamination was employed to construct strawberry safety system and determine the probability of customers being infected with NoV and HAV by contact with imported strawberries. Given frequency of strawberry trade between the United States, Australia, and China (Figure 1b), strawberries from these two countries were studied regarding the risk of virus contamination. The model was able to clarify the responsibilities of everyone involved, ensure the quality and safety of berries, and meet public's needs for food safety.

## 2 MATERIALS AND METHODS

### 2.1 Construction of critical control point system

The critical control point system was used to evaluate the hazards related to the production of a specific agricultural product, determine their production stage occurrence, and design the control mechanism. Through systematic analysis and evaluation of various factors affecting virus contamination in the strawberry supply chain, the key control point system of enteric virus represented by HAV and NoV was established.

#### 2.1.1 Construction of expert group

**FIGURE 2:**
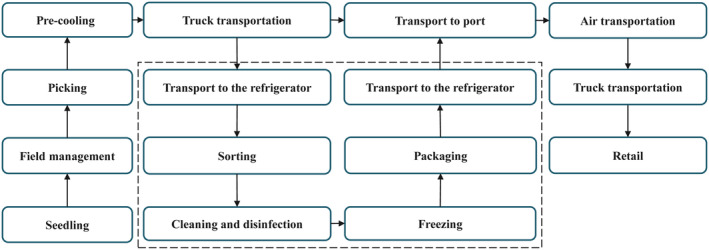
The supply chain involved in the import of strawberries. The dotted line represents the processing flow of strawberries in the processing plant.

Team members are research groups in food microbiology and foodborne disease epidemiology, and have rich experience in food safety research activities, risk assessment procedures, and food safety management systems. Through investigations, the research team established a flow chart of the production and marketing of imported strawberries (Figure 2). The research objects were divided into three kinds of strawberries, namely processed fresh strawberries (PS), unprocessed fresh strawberries (US), and frozen strawberries (FS). US are directly transported to the port by trucks after being picked. PS and FS are transported to the processing plants for processing after being picked. The difference is that FS are frozen by rapid freezing after sterilization and cleaning, while PS are not frozen. According to the information collected in the literature and the professional experience of the team members, the related activities in the strawberry supply chain were summarized and put forward control measures.

#### 2.1.2 Determination of key control points

**FIGURE 3:**
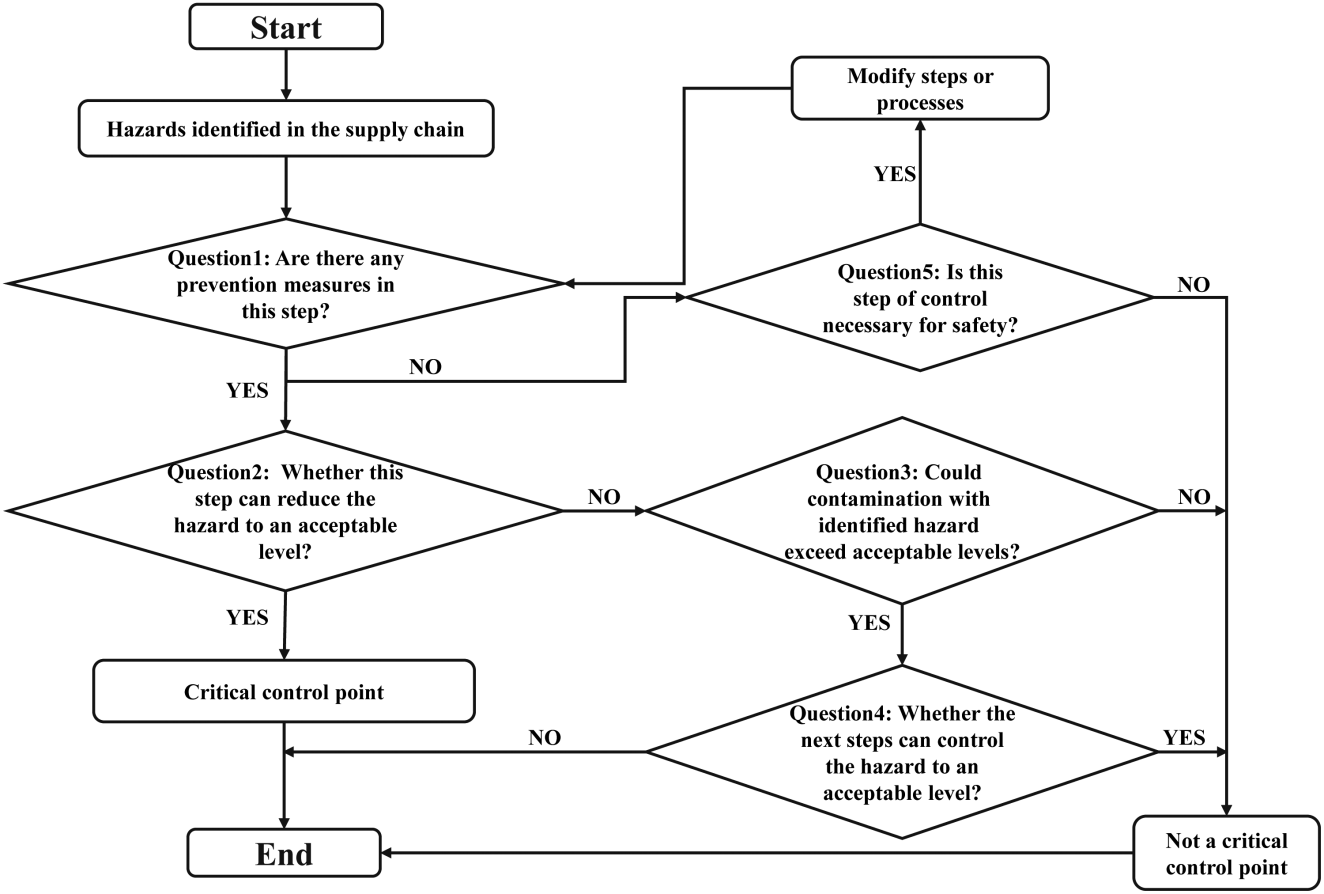
Diagrammatic representation of a decision tree for the determination of the critical control points.

According to the management status of strawberry production and marketing and the literature database, the team determined the virus contamination problem and its causes in strawberry production and marketing through a brainstorming exercise, analyzing the difficulties of strawberry supply chain supervision. A decision flow chart was used at each phase to identify potential virus contamination and whether it is a key control point (Figure 3). For the critical control points, the team developed a schedule, determining a suitably reliable control mechanism to meet the requirements of the critical limits, eliminating the risk of virus contamination in the production and marketing of imported strawberries.

### 2.2 Quantitative risk assessment model for microorganisms

The last irrigation before harvest is the main factor of virus contamination of strawberries. The risk model assumes that contamination before the harvest comes mainly from surface water, and the model considers the pollution brought in by workers in the strawberry supply chain. The model starts from field management, picking, and continues to transportation to processing facilities, washing and disinfection, freezing, transportation to port, aircraft transportation, and so on (Figure 2). Published literature data and survey data were brought in so as to maximize the capabilities of the model. The model parameters and their corresponding probability distributions are shown in Table 1. PS, US, and FS were all selected as risk assessment subjects. For PS, all the risk factors in Table 1 (except freezing) were considered, and for US, the processing sections (D16–D26) were not considered. The transportation temperature for FS after processing was lower than 0°C (Bartsch et al., 2018), so virus reduction during transportation was not considered (sections D27–D38).

**TABLE 1:** The virus exposure assessment model and a description of the variables employed.

Variable	Description	Value/distribution/calculation	Unit	References
D1	Preharvest	–	–	
D2	Initial concentrations of virus in surface water	See Figure 4	Log PDU/L	Jahne et al. (2020), Flannery et al. (2012), Ahmed et al. (2020) Osuka et al. (2018), Mok et al. (2014), Aburto‐Medina et al. (2019), Brooks et al. (2005)
D3	The amount of spray on the strawberry	Uniform (0.000888152, 0.00748)	L/berry	Miranda and Schaffner (2018)
D4	The preharvest interval between the last pesticide application and the harvesting of strawberries	Pert (0, 7, 14)	Days	Survey
D5	Virus decay rate	Normal (0.12, 0.03)	LogPDU/day	Danyluk and Schaffner (2011)
D6	Number of viruses on strawberries at harvest	10^(D2 × D3–D4 × D5)^	PDU/berry	
D7	Picking	–	–	
D8	The prevalence of the virus on the hands of worker	Beta (7, 35)	–	León‐Félix et al. (2010)
D9	The proportion of virus transferred from the product to the hands (*f*_prod_)	Beta (15.64, 41.94)	–	Bouwknegt et al. (2015)
D10	The surface area of the hand contacting strawberry (ω_touch_)	2.1	cm^2^	Verhaelen et al. (2013)
D11	Total surface area of one side of single hand (ω_hand_)	245	cm^2^	Bouwknegt et al. (2015)
D12	Surface area of the product (ω_prod_)	Normal (1064, 167)/100	cm^2^	Bouwknegt et al. (2015)
D13	The proportion of virus transferred from the hand to the strawberry (*f*_hand_)	Lognormal (−8.34, 0.58)	–	Regulation (2015)
D14	The number of viruses on the hands of workers (*N*_hand_)	D8 × Gamma (0.14, 54.6)	PDU/hand	Ortúzar et al. (2020)
D15	The number of viruses on harvested strawberries (*N*_fcross_)	Equation (1)	PDU/berry	Ortúzar et al. (2020)
D16	Transportation and processing	–		
D17	Reduction of viruses after transport to processing facilities	See Table 2	Log PDU/berry	
D18	Temperature	Pert (−1, 0, 10)	°C	Survey
D19	Time	Pert (0.00347, 0.08333, 1)	Days	Ortúzar et al. (2020)
D20	The number of viruses on strawberries at the beginning of processing	10^(Log (D15) – D17)^	PDU/berry	
D21	Virus number on strawberries after sorting	Equation (1)	PDU/berry	
D22	Virus reduction after cleaning	Normal (0.667, 0.332)	Log PDU/berry	Butot et al. (2008)
D23	Virus reduction at 100 ppm chlorine concentration	Normal (1.35, 0.24)	Log PDU/berry	Ortiz‐Solà, Abadias, et al. (2020), Predmore and Li (2011)
D24	Virus reduction after freezing	0	Log PDU/berry	Ortúzar et al. (2020)
D25	The number of viruses on strawberries after freezing	10^(Log (D21) – D22 – D23 – D24)^	PDU/berry	
D26	The number of viruses on strawberries after packaging	Equation (1)	PDU/berry	
D27	Virus reduction of strawberry during transportation from truck to port	See Table 2	Log PDU/berry	
D28	Time	ROUND (Uniform (0.5, 2), 0)	Days	Survey
D29	Temperature	Pert (−1, 0, 10)	°C	Survey
D30	Virus reduction of strawberries during airplane transportation	See Table 2	Log PDU/berry	
D31	Time	ROUND (Uniform (minimum, maximum), 0)	Days	Survey
D32	Temperature	Pert (−1, 0, 10)	°C	Survey
D33	Virus reduction after transport to distribution center	See Table 2	Log PDU/berry	
D34	Time	ROUND (Uniform (0.5, 5), 0)	Days	Survey
D35	Temperature	Pert (−1, 0, 10)	°C	Survey
D36	Virus reduction of strawberry after transport from distribution centers to catering facilities	See Table 2	Log PDU/berry	
D37	Time	ROUND (Uniform (0.5, 5), 0)	Days	Survey
D38	Temperature	Pert (−1, 0, 10)	°C	Survey
D39	The number of viruses on a single strawberry	LOG (D26)‐(D27 + D30 + D33 + D36)	Log PDU/berry	
D40	Dose–response	–	–	
D41	The number of viruses on a single strawberry	10^D39^	PDU/berry	
D42	Amount of strawberries per serving	60	g	Miranda and Schaffner (2018)
D43	The number of viruses ingested by consumers	D41 × D42	PDU/part	
D44	η	2.55 × 10^−3^	–	Miranda and Schaffner (2018)
D45	*r*	0.086	–	Miranda and Schaffner (2018)
D46	Probability of illness	1 – (1 + D43 × 0.00255)^−0.086^	–	Miranda and Schaffner (2018)

#### 2.2.1 Pollution source

Before strawberries were picked, groundwater is one of the major sources of agricultural water; surface water also account for certain proportion (Yeargin et al., 2021). Because surface water poses a great risk to fruit and vegetable crops when contaminated with foodborne pathogens (Rodrigues et al., 2020), we considered surface water in this study. The risk model assumes that the pollution before harvest is caused by viruses in surface water. Although viruses cannot reproduce in water, these viruses can remain infectious for a long time (Verhaelen et al., 2013). The detection data of NoV and HAV in surface water of the United States and Australia were selected (Aburto‐Medina et al., 2019; Ahmed et al., 2020; Brooks et al., 2005; Flannery et al., 2012; Jahne et al., 2020; Mok et al., 2014; Osuka et al., 2018). The probability distribution of virus contamination levels in surface water was obtained from the published literature data and fitting the detection data relating to viruses in surface water from these two countries with the @risk 7.6.1.

#### 2.2.2 Picking

Many pesticides have a defined preharvest interval which is the time between the last pesticide application and the harvest. During the preharvest interval, the virus will be exposed to the environment and loses viability over time. The Australian Pesticide and Veterinary Drug Administration (https://apvma.gov.au) stipulates that the preharvest interval of crops should not exceed 14 days in most cases and be at least 7 days. The United States Environmental Protection Agency (https://www.epa.gov) stipulates that the preharvest interval of strawberries is generally 14 days. Given the similarity of the collected data between the two countries, the two countries were deemed to have adopted the same safety interval in this study. The interval range for different pesticides was set to 0–14 days, considering any possible illegal operations. During the picking process, the virus from the strawberries is transferred to the hands of the workers, and the virus from the hands of the workers is transferred to the strawberries, so the contamination is reciprocal. Picking may cause cross‐contamination of NoVs between the agricultural products and the workers' hands (Bouwknegt et al., 2015), and the calculation formula used is as follows:

(1) $$N_{\text{fcross}}=N\text{harv}-f\text{prod}\;\frac{\text{ωtouch}\;}{\text{ωprod}\;}N\text{harv}+f\text{hand}\;\frac{\text{ωtouch}\;}{\text{ωhand}}\;N\text{hand}$$

where *f*_prod_ is the proportion of virus transferred from the product to the hands, *f*_hand_ is the proportion of viruses transferred from the hands to the strawberries, ω_touch_ is the hand surface area in contact with the strawberries, ω_prod_ is the surface area of the product, ω_hand_ is total surface area of one side of a single hand, *N*_hand_ is the number of viruses on the hands of workers, *N*_fcross_ is the number of viruses on harvested strawberries (sections D15, D21, and D26), and Nharv is the number of viruses on the hands of workers (sections D6, D20, and D25).

#### 2.2.3 Processing

In the model, the contamination of infected individuals in processing is considered. A cross‐contamination formula for picking was adopted for strawberries sorting and packaging. According to the team, once strawberries enter the processing facilities, they will be cleaned and disinfected. Strawberries were cleaned for 2 min and then disinfected with 100 ppm chlorine concentration for 2 min (Ortiz‐Solà, Abadias, et al., 2020). After that, FS needed to enter the rapid freezing process. Freezing had no significant effect on the infectivity of viruses, and the virus particles kept the integrity of their structures and genomes after several freeze–thaw cycles (Ortúzar et al., 2020).

#### 2.2.4 Transport and storage

It was assumed that US and PS needed to be transported by truck from the storage place to the port, then by plane to a Chinese port, by truck from the port to the distribution center, and finally by truck from the distribution facilities to the catering facilities. According to the actual survey situation, the transportation intervals from the United States and Australia to China were set to 2–3 and 0.5–3 days, respectively. One of the main factors that affect the virus contamination of strawberries is the reduction of viruses that occur under various temperature conditions over some time. The study used a model (Table 2; Bertrand et al., 2012) to simulate the inactivation of NoV and HAV at an ambient temperature above 0°C, and the number of viruses was assumed to remain unchanged below 0°C (Ortúzar et al., 2020).

**TABLE 2:** Storage and transportation pollution models of virus for temperature above 0°C.

Variable	Description	Value/distribution/calculation	Unit
−*X*_TFL_	Mean time of first log reduction	NoV: 1.8 – T × 0.03 HAV: 2.0 – T × 0.03	Log (day)
*S*_TFL_	Standard deviation of the time of first log reduction	NoV: $\sqrt{0.37+6.4\times 10-4T+1.4\times 10-5T2}$ HAV: $\sqrt{0.31+6.1\times 10-4T+1.4\times 10-5T2}$	Log (day)
*T*	Temperature of transportation and storage	NA	°C
LogTFL	Time of first log reduction	Normal (−X_TFL_, S_TFL_)	Log (day)
μ	Virus reduction per day	10^−logTFL^	LogPDU/day
t	Time of transportation and storage	NA	Days
Log (Ni)	Virus reduction during storage and transportation	μ × *t*	LogPDU/berry

*Note*: The parameters and equations were adopted from Bertrand et al. (2012).

#### 2.2.5 Preparation and consumption of catering facilities

The weight of a single strawberry was estimated as 15 g (Li et al., 2005). It was assumed that strawberries would not be heated before eating. We set the daily fruit intake as the daily strawberry intake of Chinese residents (Table 3). A scenario analysis was carried out based on the strawberry intake of residents, with the calculation of possible poststrawberries intake exposure to viruses by different age groups residents.

**TABLE 3:** The recommended daily fruit intake of Chinese residents was set as these amounts.

Age group	Fruit intake (g/day)	
	Men	Women
2, 4	34	38.1
4, 7	32.2	37.2
7, 11	38.5	39.5
11, 14	42.4	43.6
14, 18	34.9	47.5
18, 45	30.7	45.2
45, 60	30.8	40.1
>60	30.3	30.1

*Note*: The data come from the Fifth China Total Diet Study.

#### 2.2.6 The dose–response model

The purpose of a dose–effect assessment was to determine the relationship between the exposure intensity to virus and the severity of harmful health effects encountered. An existing dose–response model for the probability of illness (Pi) among the infected population was used. The values of η and *r* in the model were estimated at 2.55 × 10^−3^ and 0.086, respectively (Miranda & Schaffner, 2018). A single strawberry consumption of 60 g was considered, though it will vary according to a given situation.

(2) $$P_{i}=1–{\left(1+\eta \times \text{dose}\right)}^{-r}$$

#### 2.2.7 Simulation

The extracted data were entered into a spreadsheet (Table 1), and each evaluation scheme was iterated 100,000 times by using an external program @risk7.6.1 of the spreadsheet, and Monte Carlo simulation was performed to explore the variation degree of each parameter and reveal the influence degree of each factor on NoV and HAV pollution in edible strawberries.

## 3 RESULTS

### 3.1 Critical control point system

#### 3.1.1 Harm analysis of virus in the strawberry supply chain

According to the strawberry supply chain (Figure 2), the expert team proposed the possible ways of causing virus contamination in the production and marketing of strawberries (Ortiz‐Solà, Viñas, et al., 2020; Wu et al., 2020). Here each step in the strawberry supply chain was analyzed to determine critical control points in the whole process based on the expert team's suggestions (Table 4).

**TABLE 4:** Harmful analysis of virus contamination in the strawberry supply chain.

Process	Associated activities	Control measures	The questions of decision tree					Is it a key control point
			Question 1	Question 2	Question 3	Question 4	Question 5	
Seedling	Planting. Cultivation of sound seedling. Spraying of pesticides.	No	No	–	–	–	No	No
Field management in fruiting stage	Irrigation. Spraying of pesticides.	Use of drinking water or clean water. Regular monitoring of virus concentration in water.	Yes	Yes	–	–	–	Yes
Picking	Manual picking. Packing and storage.	Worker receive proper hand washing training. Regular disinfection of workplace.	Yes	No	Yes	Yes	–	No
Precooling treatment	Vacuum precooling. Cold storage.	Regular disinfection of workplace. Precooling equipment shall be disinfected regularly.	Yes	No	No	–	–	No
Sorting	Conveying by conveyor belt. Manual sorting.	Regular disinfection of workplace. Worker receives proper hand washing training.	Yes	No	Yes	Yes	–	No
Cleaning and disinfection	Conveying by conveyor belt. Cleaning and disinfection.	Use of drinking water or clean water. Use of effective disinfectants or disinfection methods.	Yes	Yes	–	–	–	Yes
Freezing	Conveying by conveyor belt. Quick‐freezing.	Equipment shall be disinfected regularly. Regular disinfection of workplace.	Yes	No	No	–	–	No
Packaging	Conveying by conveyor belt. Personnel packaging.	Regular disinfection of workplace. Personnel standard operation.	Yes	No	No	–	–	No
Transport to refrigerator	Personnel handling. Storage.	Regular disinfection of workplace.	Yes	No	No	–	–	No
Transport to port	Personnel handling. Storage.	Regular disinfection of workplace.	Yes	No	No	–	–	No
Air transportation	Personnel handling. Storage.	Regular disinfection of workplace.	Yes	No	No	–	–	No
Truck transportation	Personnel handling. Storage.	Regular disinfection of workplace.	Yes	No	No	–	–	No
Retail	Storage. Consumers choose strawberries.	The products are well packed. Regular disinfection of workplace.	Yes	No	No	–	–	No

Most virus outbreaks related to fresh fruits are caused by irrigation with polluted water in the field (Prez et al., 2018). As strawberries grow, the water used to irrigate them is susceptible to contamination, such as human and animal waste. Studies have shown that the contact between irrigation water and strawberries causes virus contamination in strawberries, which is the main type of pollution to occur before harvest (Mok & Hamilton, 2014). Farmers may mix pesticides in nonpotable water, increasing the risk of virus contamination of the fruits. Therefore, field management is the key control point. In the picking and sorting stage, viruses maybe spread to the strawberries by staff due to problems of hand hygiene or cross‐infection through the handling of contaminated strawberries (Gao et al., 2019). Virus contamination in sorting and packaging stage can be controlled by disinfection and washing.

Disinfectants in water can remove or reduce pathogens on strawberries. Therefore, cleaning and sterilization are the key control points in this study. In the supply chain, cold chain integrity management is an important factor to satisfy food quality and safety requirements (Mukama et al., 2020). Potential risks of virus contamination of strawberries derived from transportation environmental pollution are small because strawberries are packed before transportation which prevents direct contact with workers and the environment. Therefore, transportation was not considered to be a critical control point.

In this study, the key control point system was established to analyze the virus contamination factors in the strawberry supply chain, and finally the two steps of field management and disinfection were determined as the key control points (Table 4).

#### 3.1.2 Safety management system

According to the key control points in strawberry production and marketing, a safety management system was established to ensure product quality and safety (see Table 5). Water quality monitoring was the key point to ensure the health safety of strawberries. According to scientific reports, the minimum dose for both NoV and HAV infection is 10 virus particles (Atmar et al., 2013). In the field management, the content of virus per liter of irrigation water is required to be less than the minimum infection dose of 10 virus particles. Therefore, more precise, convenient, and rapid virus detection methods should be considered such as paper‐based colorimetric methods (Sun, Cao, et al., 2021) and reverse transcription droplet digital polymerase chain reaction (RT‐ddPCR) measurements (Sun, Chen, et al., 2021). Disinfection and washing processing are the key control points in strawberry production and marketing. While ensuring safety, improving the efficacy of disinfection and washing was the final goal. Through reference analysis (Ortiz‐Solà et al., 2021), the key limits of different disinfectant concentrations and treatment times were determined.

**TABLE 5:** Key control system of quality and safety in strawberry supply chain.

Critical control point	Critical control limit	Monitoring object	Monitoring method	Measures	Recording	Verification
Field management in fruiting stage	Virus in irrigation water is less than the minimum infection dose 10 PDU/L. The safe interval before picking is greater than 7 days.	Irrigation water. Strawberries after harvest.	Real‐time monitoring of irrigation water by virus detection standard. Strawberries are harvested after a safe interval.	Strawberries shall not be planted if the irrigation water test result does not meet the standard. Strawberries should not be harvested during the safety period.	Monitoring report records of irrigation water and safety interval should be established	Water resources around farms should be regularly tested for viruses. Farm operation procedures should be checked regularly. Producers' knowledge of virus protection should be tested regularly.
Cleaning and disinfection	The concentration of disinfectant is 80 ppm peracetic acid, 5% hydrogen peroxide, 25% organic acid, and 100 ppm sodium hypochlorite, respectively. The cleaning and disinfection time of strawberry is more than 2 min.	Disinfectant. Water. Strawberry.	Quality inspection of washing water. Monitoring the concentration of disinfectant. The staff regularly checks strawberries for viruses.	When the strawberry sampling inspection is unqualified, the staff needs to picket the cause of the accident and disinfect the strawberry again.	Workers establish virus detection information in washing water. The staff set up records of strawberry disinfection and cleaning and sampling inspection.	The processing procedures of enterprises and the ability of operators should be tested regularly.

### 3.2 Quantitative risk assessment of viruses

The water pollution‐related factors in key control points should be considered in building the QMRA model. On the other hand, the health factors of workers are also considered in the model.

#### 3.2.1 Distribution of the virus concentration in surface water

**FIGURE 4:**
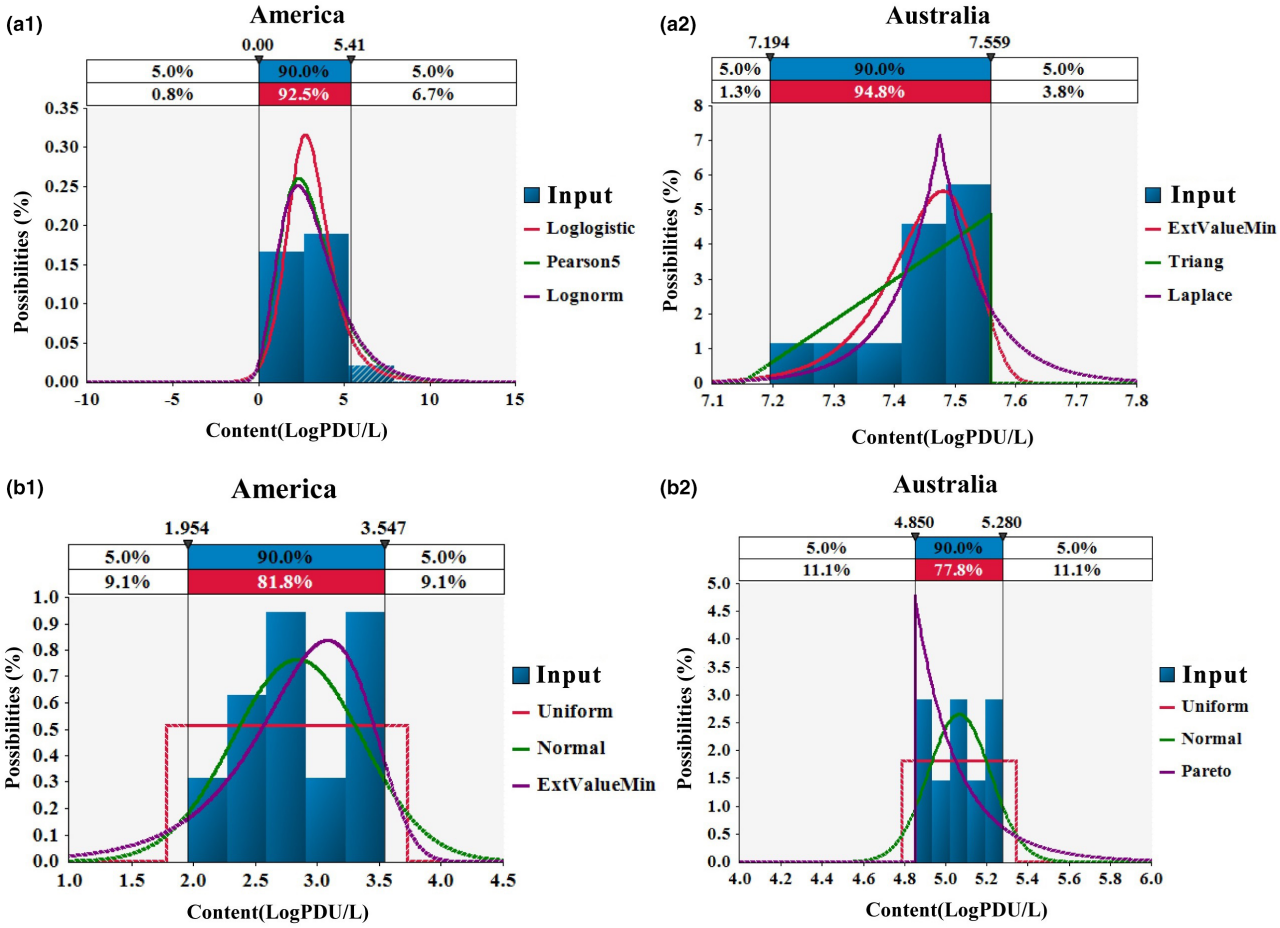
The fitted distribution curves of virus concentration in the surface water. a‐1, a‐2 represent NoV; b‐1, b‐2 represent HAV.

Virus contamination data in surface water from the United States and Australia were collected by survey research methods. The probability distribution function was used to simulate the virus contamination level in surface water of two countries. @Risk software automatically generates distribution according to the input data and arranges the fitting distribution results in sequence according to AIC (Akaike information criterion; Figure 4). The distribution of NoV pollution in surface water of the America and Australia is LogLogistic (−3.772, 6.6985, 8.3455) and ExtValueMin (7.4802, 0.066393), respectively. The distribution of HAV pollution in surface water of the America and Australia is Uniform (1.7773, 3.7239) and Uniform (4.78857, 5.34143), respectively.

#### 3.2.2 Analysis of virus contamination level in a single strawberry

**FIGURE 5:**
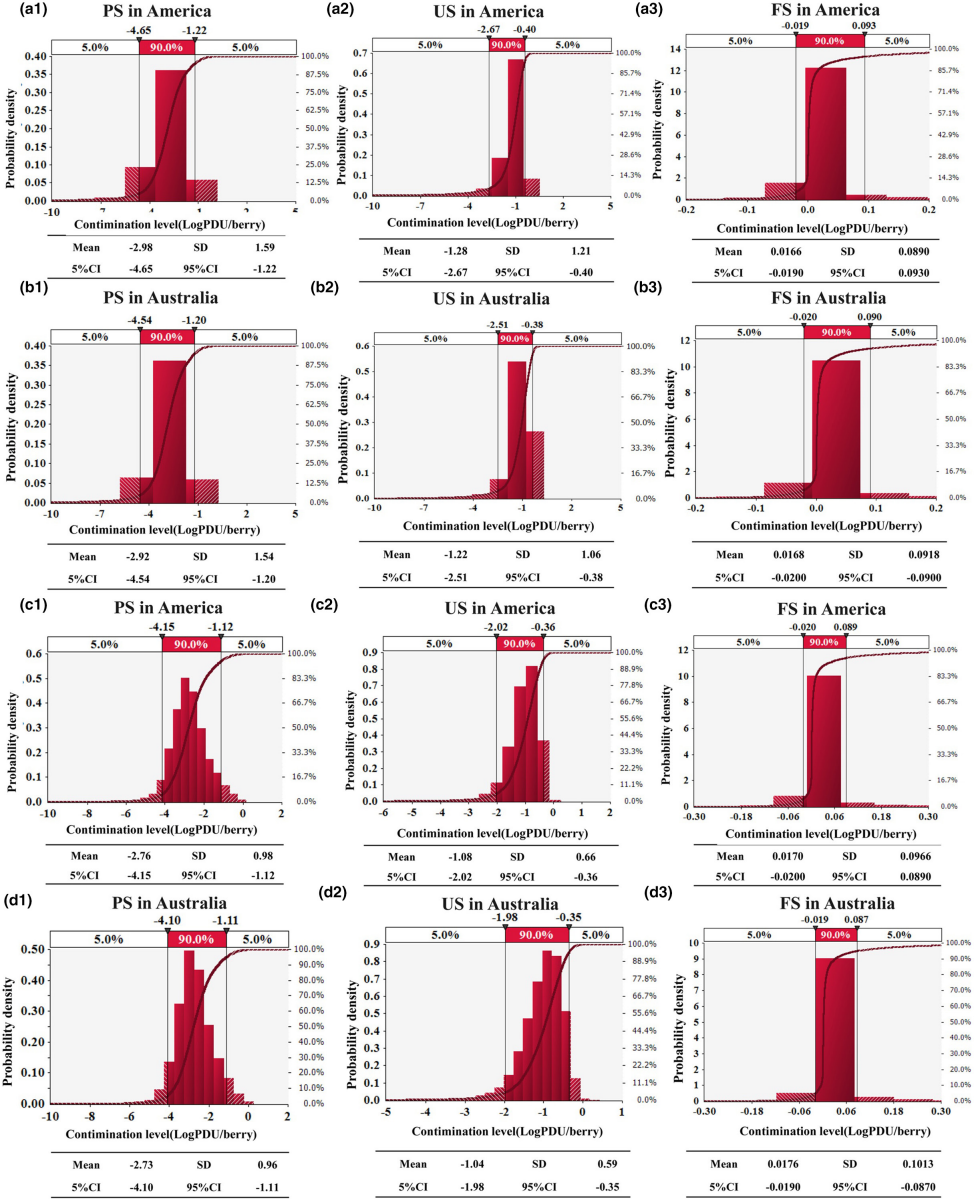
The possibility distribution of NoV and HAV contamination levels in a single strawberry. The weight of strawberry is 15 g; a‐1, a‐2, a‐3 and b‐1, b‐2, b‐3 represent NoV; c‐1, c‐2, c‐3 and d‐1, d‐2, d‐3 represent HAV. PS means processed fresh strawberries, US means unprocessed fresh strawberries, and FS means frozen strawberries.

As shown in Figure 5, the order of the average contamination level of NoV in strawberries was as follows: PS_America_ (−2.98 LogPDU/berry) < PS_Australia_ (−2.92 LogPDU/berry) < US_America_ (−1.28 LogPDU/berry) < US_Australia_ (−1.22 LogPDU/berry) < FS_America_ (0.0166 LogPDU/berry) < FS_Australia_ (0.0168 LogPDU/berry). Compared with the same kind of strawberries, the pollution level of NoV in Australian strawberries is higher than that in American strawberries. This result is consistent with the information published in the two national institutions. The average annual number of NoV infections reported by the Center for Disease Control and Prevention (https://www.cdc.gov/) in the United States is 601,378. The Australian Ministry of Health (https://www.health.gov.au/) reported that, on average, 1,800,000 Australians are infected with NoVs every year.

The statistics shows that the main reasons for the outbreak of hepatitis A are the health problems of the homeless and the virus contamination of food. There are no outbreak cases of homeless groups in Australia. The average annual number of hepatitis A cases in the United States (https://www.cdc.gov/) excluding the homeless is 3025. The annual average number of hepatitis A cases in Australia (http://www.health.gov.au/) is 299. The population density of the United States and Australia (httpsww.worlddata.info/) is 33 and 3, respectively. The severity of the food‐related hepatitis A outbreak was expressed by the ratio between the average number of hepatitis A cases to the population density, which was 90.6 in America and 91.2 in Australia. Therefore, the outbreak degree of food‐related hepatitis A is consistent with the pollution results calculated in this study.

**FIGURE 6:**
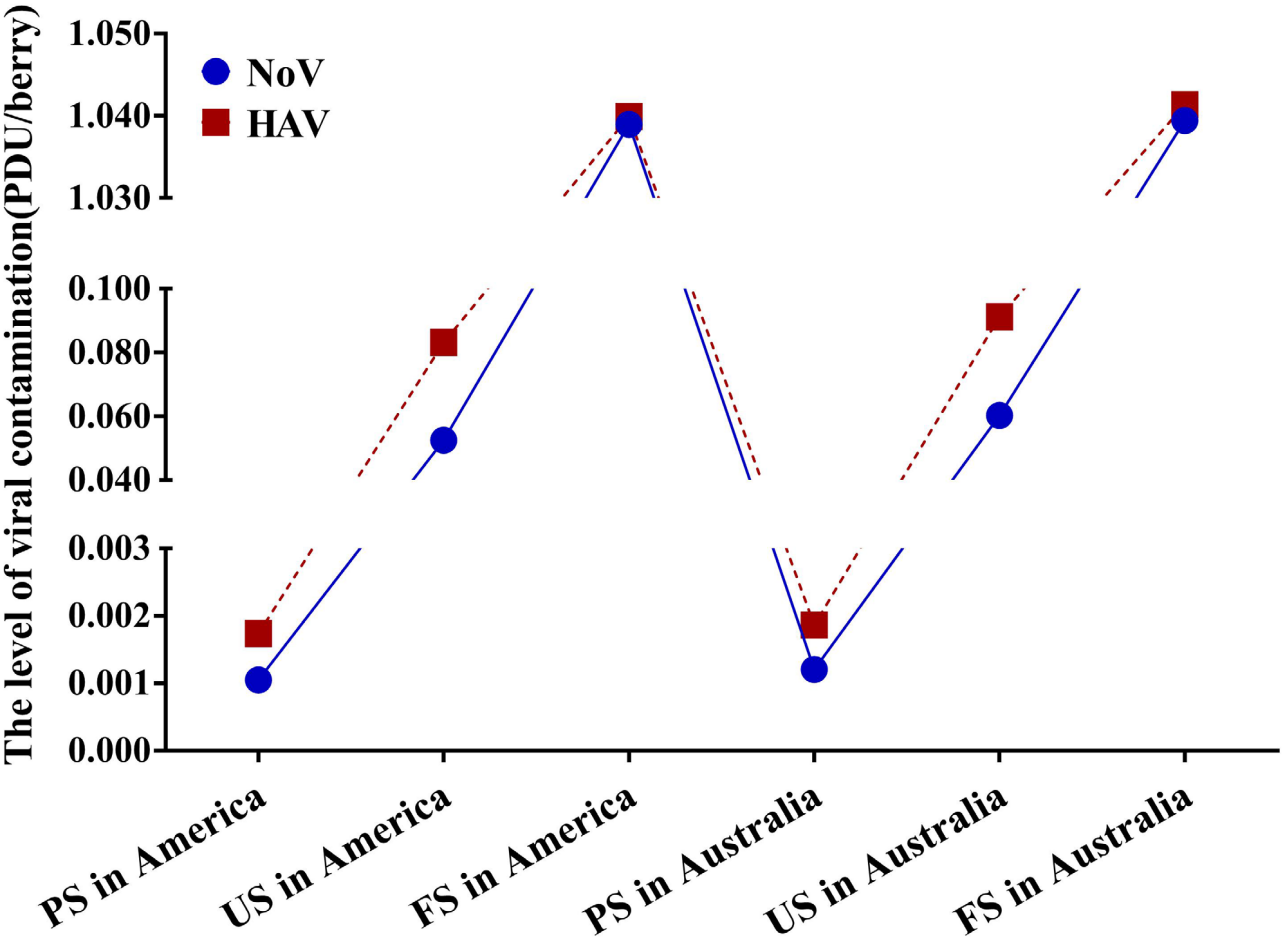
Mean levels of NoV and HAV contamination in strawberries.

It was found that the amount of virus attached to the surface of PS was less due to the cleaning and disinfection steps. After processing, FS were stored below 0°C, and the number of viruses attached to the surface of strawberries remains unchanged (Gao et al., 2019), resulting in a large number of viruses on FS (Figure 6). At the same time, the results (Figure 6) show that the pollution level of the HAV in strawberries in the United States and Australia is higher than that of NoV. However, the number of hepatitis A cases is lower than that of NoV is because the coverage rate of hepatitis A immunization is wide in the national childhood immunization program (Garcia Garrido et al., 2019). Inactivated hepatitis A vaccines have highly immunogenic in healthy, reducing the incidence of hepatitis A (Andani et al., 2021). NoV has greater genetic diversity, so it is harder to develop related vaccines and there is no licensed vaccine at present (Hasing & Pang, 2021).

**FIGURE 7:**
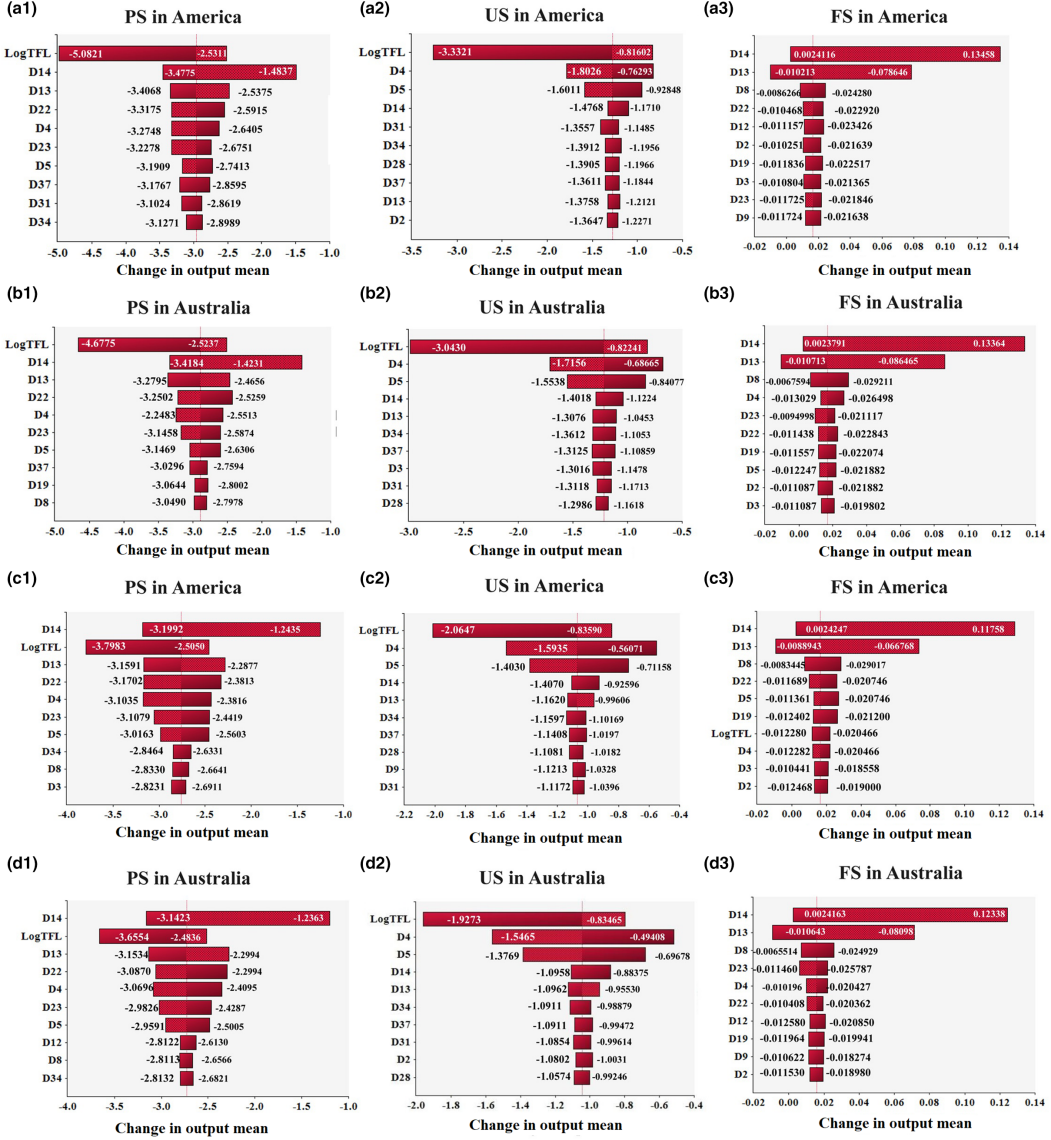
Sensitivity analysis of factors affecting the final concentration of NoV and HAV in a single strawberry. a‐1, a‐2, a‐3 and b‐1, b‐2, b‐3 represent NoV; c‐1, c‐2, c‐3 and d‐1, d‐2, d‐3 represent HAV.

Sensitivity analysis is a method for analyzing the statistical correlation between input and output values in microbial quantitative risk assessment. Sensitivity analysis of the factors affecting the final virus concentration in a single strawberry was carried out (Figure 7). The main factors affecting the concentration of virus on PS are the time of first log reduction (LogTFL), the number of viruses on the hands of workers (D14), and the proportion of viruses which can be transferred from the workers' hands to the products (D13), when exposed. The main factors affecting the virus concentration on US are the time of first log reduction (LogTFL), the preharvest interval between the last pesticide application and the harvesting (D4), and the virus decay rate (D5). The main factors affecting the virus concentration on FS are the number of viruses on workers' hands (D14), the proportion of viruses which can be transferred from workers' hands to the products (D13), and the prevalence of viruses on workers' hands (D8).

It was found that possible contamination from workers in strawberry production and marketing is another main factor affecting the virus concentration in strawberries because they are involved in picking, sorting, and packaging. In addition to human contamination, the virus viability has become a major factor affecting the virus concentration in strawberries. The main variable in the formula concerning the inactivation time of first log reduction is the temperature of transportation and storage. It indicates that the transportation and storage temperatures are particularly important to the pollution level of virus in strawberries. Low temperatures will ensure the fresh quality of the food, but at the same time, a low temperature will also lead to the persistence of the virus on strawberries (Mukama et al., 2020). Therefore, it is the recommended management strengthening during the processes of strawberry production and picking to avoid virus contamination of strawberries before transportation.

#### 3.2.3 Scenario analysis

**FIGURE 8:**
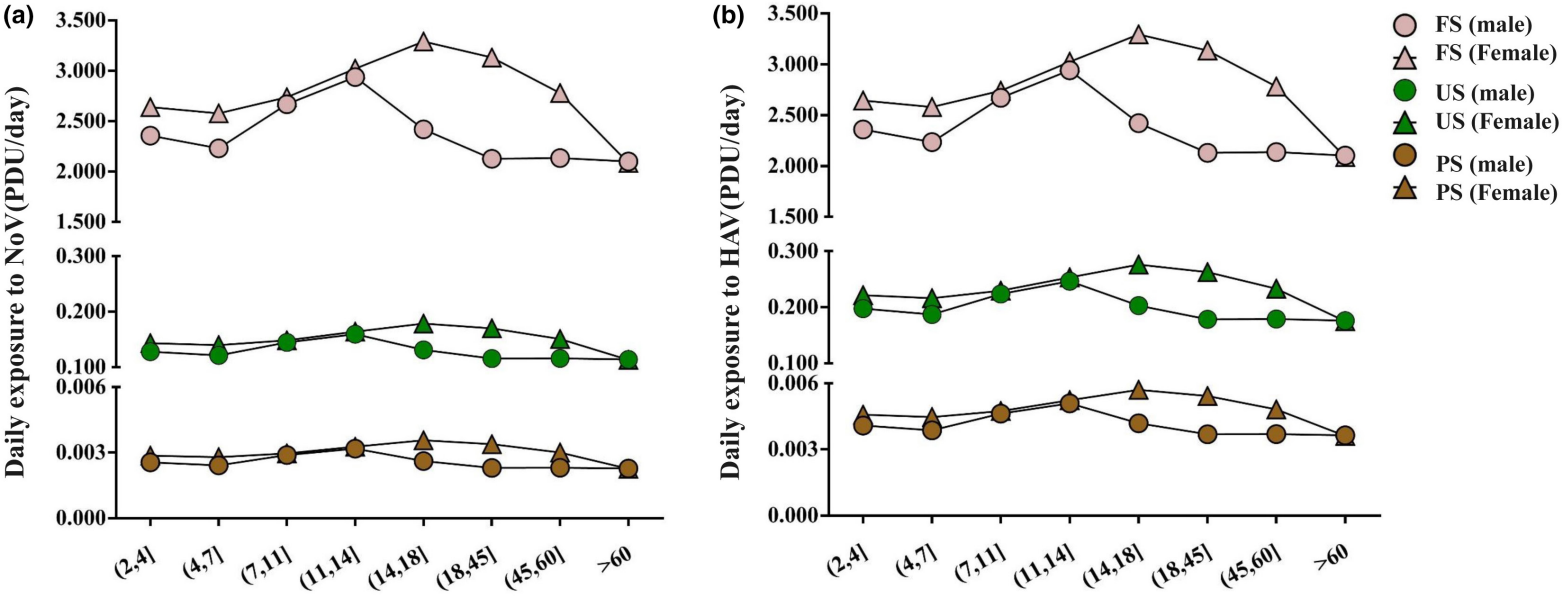
Residents' daily exposure to virus derived from strawberries when separated into different age groups. a represent NoV; b represent HAV.

The model set the daily fruit intake of Chinese residents as the daily strawberry intake of Chinese residents. The residents' strawberry intake combined with the average virus contamination level of strawberries from the United States and Australia allowed calculation of the virus exposure in Chinese residents of different age groups. It was found that the exposure to the virus by eating imported strawberries was less than the minimum infection dose of 10 PDU (Figure 8). By comparing different age groups, boys aged 11–14 years and girls aged 14–18 years have high daily exposure to viruses. The main reason is probably that these two age groups consume more strawberries daily (Figure 8).

#### 3.2.4 Dose–response

**FIGURE 9:**
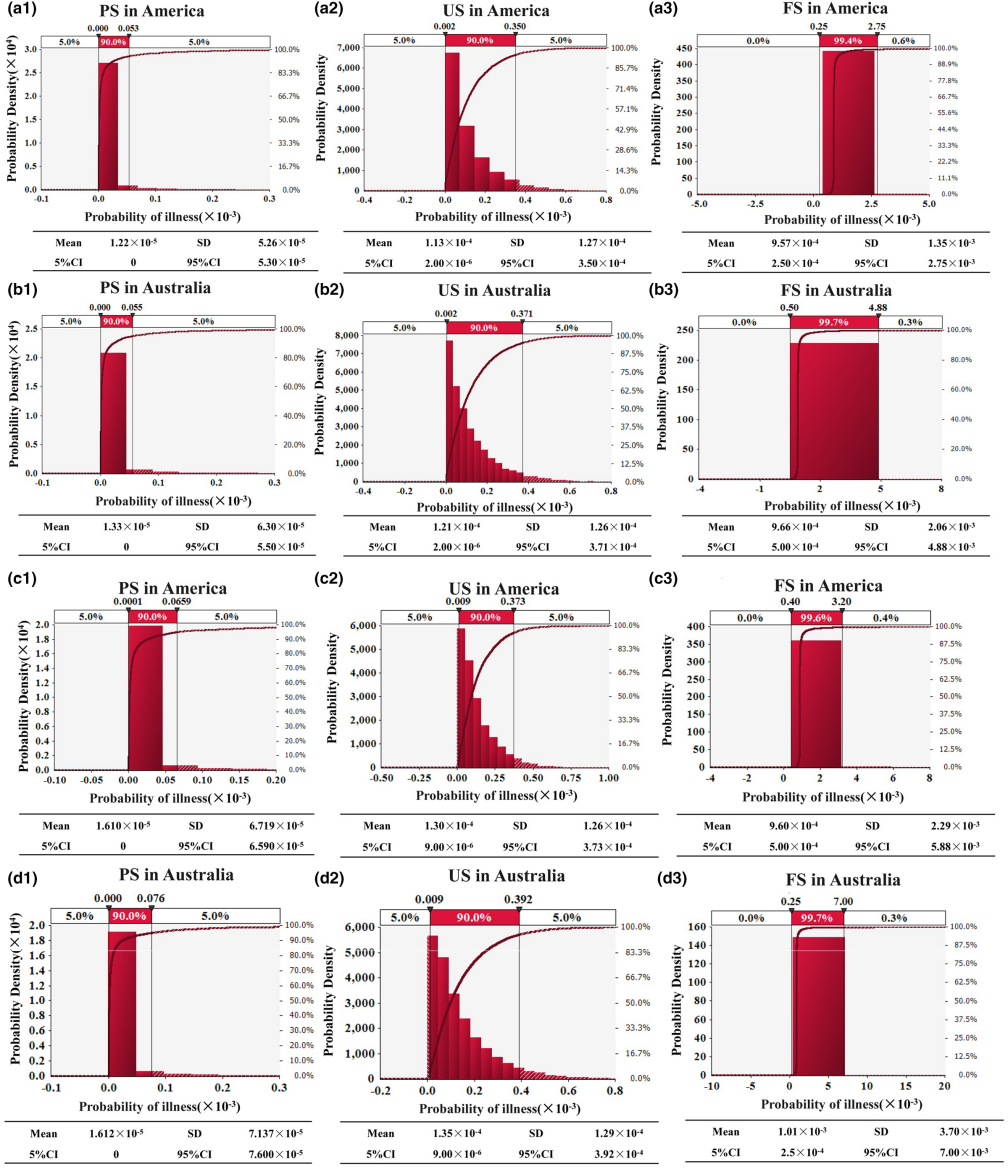
The distribution of probabilities for virus infections among the population. a‐1, a‐2, a‐3 and b‐1, b‐2, b‐3 represent NoV; c‐1, c‐2, c‐3 and d‐1, d‐2, d‐3 represent HAV.

**FIGURE 10:**
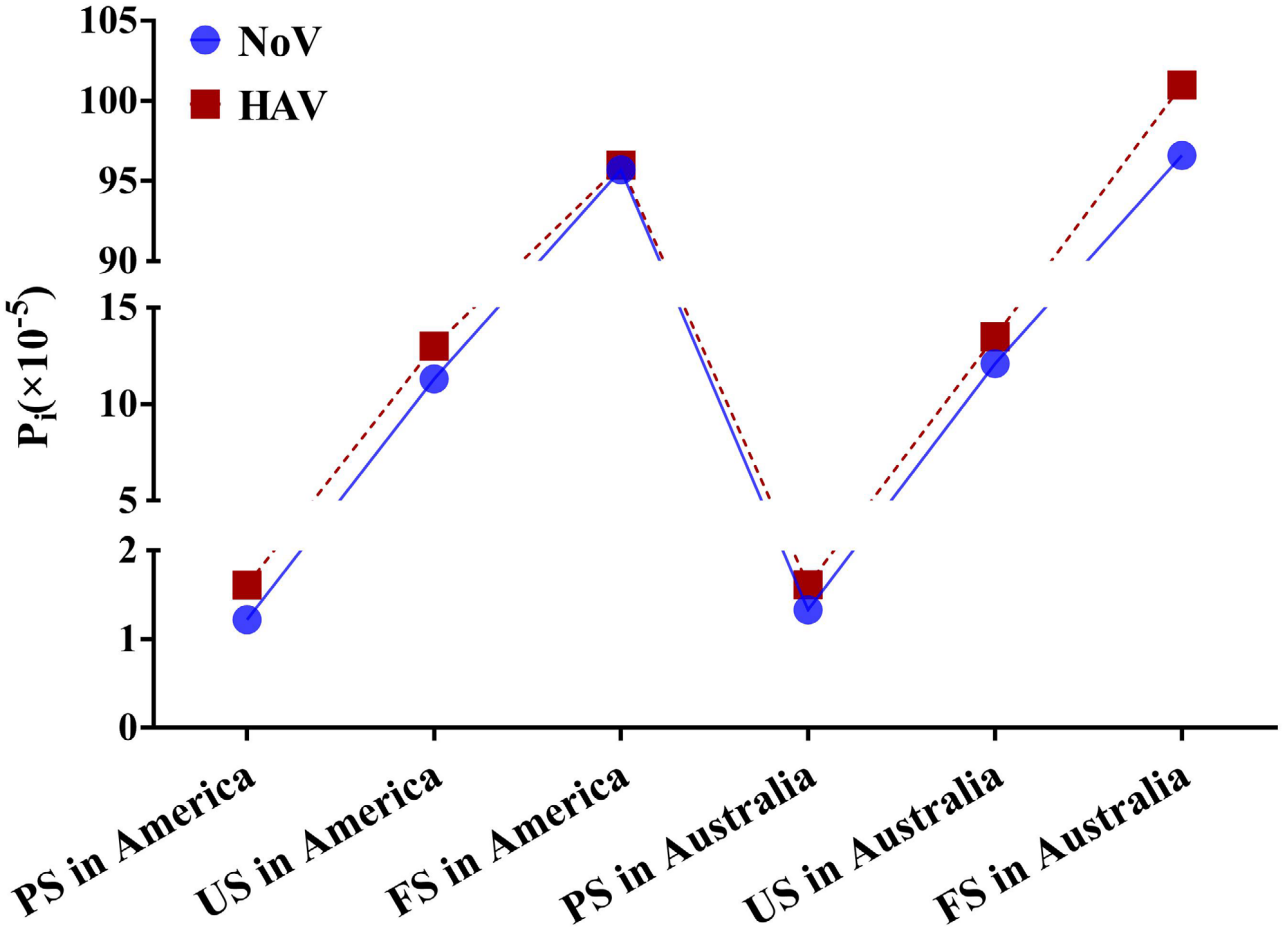
Average probabilities for NoV and HAV infections among the population.

The infection probabilities (Figure 9) of NoV and HAV after eating US is nine times and eight times that after processing, respectively. Fresh strawberries, after strict processing protocols, have a lower NoV infection rate. Increasing the cleaning and disinfection processes without causing increased damage to the strawberries can reduce the virus contamination due to fresh strawberries. The probability of NoV and HAV infection among residents after eating FS is eight times and seven times that of residents after eating US (Figure 10), and 75 times and 61 times that of residents after eating PS, respectively. It is obvious that the risk of viral foodborne diseases caused by FS is higher. The results of this study are consistent with those reported previously (Bartsch et al., 2018).

#### 3.2.5 Risk assessment system

**FIGURE 11:**
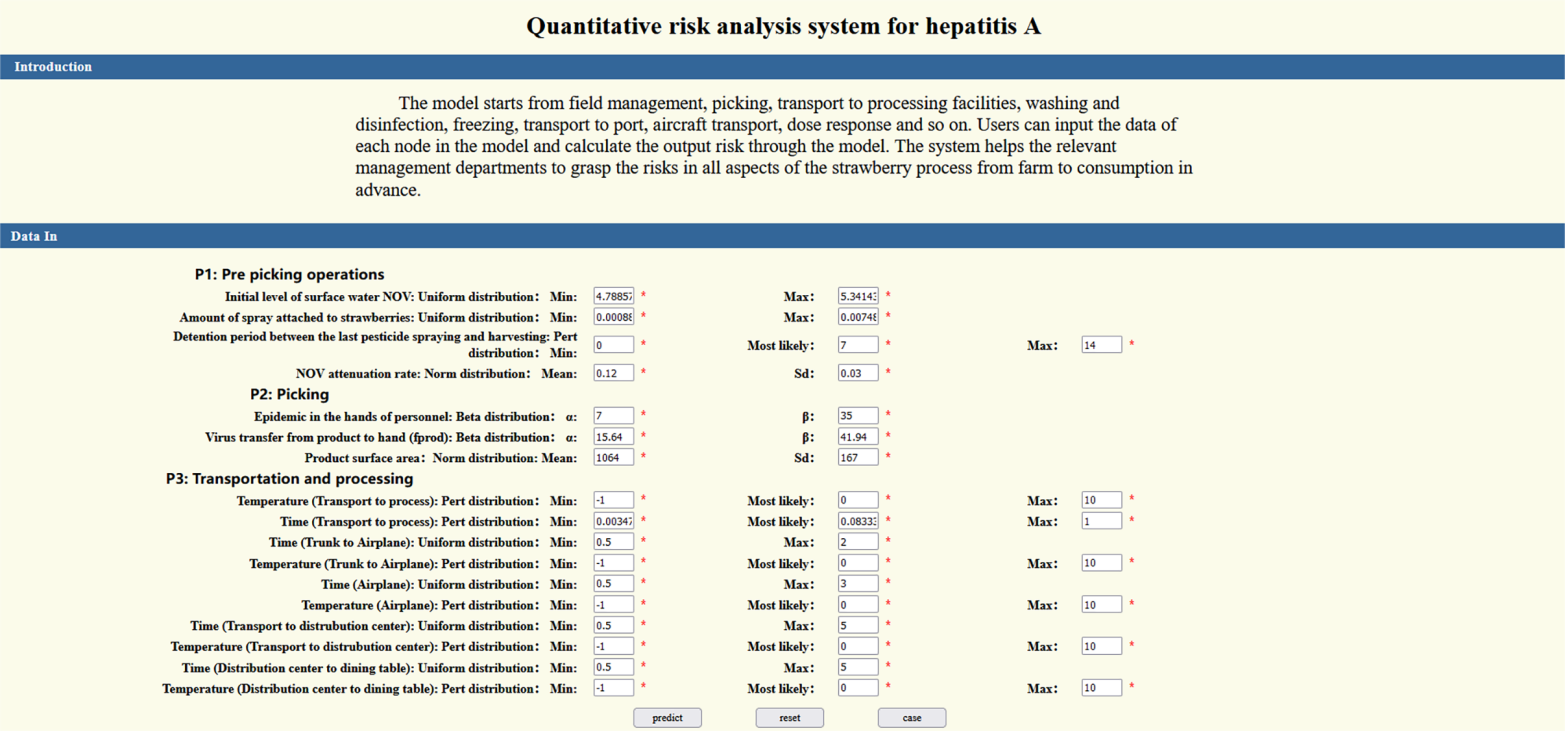
The operating interface of the risk assessment system.

Based on B/S (browser/server) architecture, this section adopts Java EE and MVC (Model–View–Controller) models to design and implement a pathogen quantitative risk assessment system. The Model is used to store and process data, the View is used in the application to realize the visualization of risk data, and the Controller is used in the application to deal with the interaction of risk analysis users. The MVC pattern is used to identify the client and server components in the Web‐based online system, and the user interaction information and interface are modified and formulated. The platform realizes the information of virus damage in the production and marketing chain of strawberry, can report the potential risk of pathogen contamination in strawberry early, improve the efficiency of risk assessment, and has certain application value. Users can through the website links (http://1.15.226.101:8080/strawberryVirus/) into the main operating system interface (Figure 11), the application system will accept calculation requests, and call the model parameter data provided by the user to calculate the final output prediction results.

## 4 DISCUSSION

NoV and HAV are the main pathogens causing gastroenteritis and hepatitis (Takahashi et al., 2018; Yao et al., 2020). Strawberries are easily polluted by viruses during production and processing, making consumers face real risks. The study by Cook et al. (2021)) found detection of HAV in strawberry samples associated with the famous 1997 outbreak of foodborne hepatitis in the United States. The detection of HAV demonstrates the persistence of this virus under freezing conditions and the ability of RT‐PCR‐based methods to identify pathogenic organisms even in long‐term stored samples. According to the literature and the actual strawberry supply chain, put forward by the expert group, a critical control point system was established to analyze the potentially dangerous situations in each work process and formulate control measures to prevent them. The main factors of virus contamination in strawberries were water resources and human contamination, and the key control points were field management, cleaning, and disinfection. Through the investigation and analysis of these two steps, some food safety protection measures were suggested, which were also applied in the schedule of the critical control point system.

Strawberries from the United States and Australia are the leading sources of China's imported strawberries. The QMRA model was used to assess the risk of virus contamination of strawberries in these two countries and the probability risk of NoV and HAV infection among Chinese consumers by eating imported strawberries. Surface water in the United States and Australia was used as a source of virus contamination in the strawberry supply chain. Studies have shown that the probability of virus infection among Chinese consumers by eating strawberries from America was less than from Australia. The probability of virus infection among consumers by eating US was nine times higher than consuming them after processing. Fresh strawberries after strict cleaning and disinfection were safer than fresh strawberries directly sold after picking. Compared with fresh strawberries, imported FS carried more viruses because they were generally stored below 0°C for an extended time. The QMRA model integrates the data of various virus contamination factors encountered in the strawberry supply chain and was used to judge the degree of final exposure of humans to viruses and evaluate the potential harm of these viruses to human health. Sensitivity analysis of potential pollution points shows that managers should carry out standardized management for workers. The managers in the strawberry supply chain should strictly abide by the work environment protocols and personal hygiene management requirements stipulated by the Food Standards Agency (www.food.gov.uk/safety‐hygiene/norovirus) and the Centers for Disease Control and Prevention (www.cdc.gov/infectioncontrol/guidelines/norovirus). Government departments should conduct management and sampling inspections on their strawberry markets. The results of strawberry detection should meet the requirements of DBS13/001‐2015 (Regulation, 2015) and GB 4789.42‐2016 (Regulation, 2016) for NoV detection in national foods. The retailers and regulatory authorities should jointly regulate food safety.

Combining the critical control point system with the QMRA model, the study can effectively determine the virus contamination factors in strawberry production and marketing, ensure the quality and safety of strawberries, and provide key quality control strategy references for managers. Our study shows that NoV and hepatitis A in many berries originate from water and food handlers, which are supported by some investigations in Europe, North America, Asia, Australia, Africa, and South America (Gonzales‐Gustavson et al., 2019). Our study considered China's main import countries, from the website (https://oec.world/) to query the trade of strawberries, we can find that the United States and Australia are closer to China's strawberry trade and other countries are not necessarily in our main trade objects. In less developed countries, there may be more people infected with NoV, so quantitative risk assessment models can be built for more less developed countries in the future to provide a virus contamination warning platform. Strawberry production and health management of workers need water resources, so it is important to understand the fate of viruses in water and wastewater.

This work provides a preliminary but relatively comprehensive and flexible framework for estimating general data. The virus contamination of fresh strawberries and FS was determined by considering the influence of all the operating conditions. Due to various limitations in data availability, we encountered challenges in obtaining a complete dataset that would accurately reflect the entire landscape of strawberry contamination. In light of these challenges, we have taken a proactive approach to address the limitations of our dataset. Recognizing that the absence of complete data could potentially impact the clarity of the problem's current situation, we have turned to simulation and modeling techniques to bridge the gap. Specifically, we have conducted risk assessments in three distinct scenarios: PS, US, and FS. These simulations have been grounded in different distribution models that allow us to extrapolate and analyze potential contamination risks under various conditions. Furthermore, we acknowledge that our current model parameters are based on available literature data and assumptions. However, we are fully committed to refining and adapting our model once additional, more comprehensive data become available. We believe that the model's flexibility allows us to adjust its parameters to align with real‐world data, which will contribute to more accurate risk assessments in the future. To enhance the accessibility and timeliness of our risk assessments, we have integrated a quantitative risk assessment online platform. This platform not only facilitates real‐time risk forecasts, but also serves as a mechanism for rapid adaptation and parameter adjustment as new data emerge. Strawberries imported from other countries to the United States and Australia and reimported to China have the potential for indirect contamination, and we will take this into account in the future. At present, PCR technology has developed rapidly, such as Fraisse et al.'s (2017)) study using a digital RT‐PCR method to quantify hepatitis A and NoV in soft berries. The model does not consider the prevalence of PCR, and relevant nodes can be set in future models to optimize the model. The reliability of microbial risk estimation needs to be further improved which can be done by refining the parameterization of identification model variables. The limited information regarding virus concentration in pollution points will affect the uncertainty of estimates regarding virus concentration. At present, we have temporarily collected these literatures, which may affect the stability and accuracy of the model. If there are any updates to the literature data in the future, we will consider including more reliable literature data. We will continue to update our model in the future and collect related data. Future research is needed whereby more sample data must be collected to clarify uncertainties of the modeling.

## 5 CONCLUSIONS

In this study, we investigated contamination risk of NoV and HAV in imported strawberries through qualitative and quantitative analyses across the supply chain. Employing a critical control point system, we identified field management and disinfection as pivotal in minimizing virus risks. Our research adhered to rigorous hygiene standards while contributing novel insights. Based on the critical control point system, the QMRA model was explored which revealed distinct contamination levels based on strawberry origin, aligning with authoritative agricultural data which showed that the contamination levels of virus in strawberries from Australia were higher than those from the United States, consistent with the information displayed by the agriculture departments of the two countries. We highlighted the disparity in safety between carefully processed and directly picked fresh strawberries, emphasizing the importance of postharvest measures. Notably, our study unveiled heightened virus persistence on FS, underlining the need for tailored handling during freezing. Our pioneering risk assessment early warning platform equips researchers and authorities with predictive tools for timely interventions.

Overall, our research advances food safety understanding, offering practical risk management strategies while enhancing the safety and quality of strawberries across the supply chain.

## AUTHOR CONTRIBUTIONS

**Junjie Zhong:** Writing – original draft (equal). **Yunfeng Yang:** Visualization (equal). **Hui Zhang:** Validation (equal). **Shuwen Zhang:** Validation (equal). **Xiaosheng Qu:** Validation (equal). **Qin Chen:** Supervision (equal). **Bing Niu:** Project administration (equal); writing – review and editing (equal).

## FUNDING INFORMATION

This study was supported by the Shanghai Agriculture Project (19391901500) and the National Key Research and Development Program of China (grant nos. 2022YFC2601200, 2022YFF0600007).

## CONFLICT OF INTEREST STATEMENT

The authors declare that they have no conflicts of interest.

## Data Availability

The data that support the findings of this study are available on request from the corresponding author.

## References

[fsn33721-bib-0001] Aburto‐Medina, A. , Shahsavari, E. , Salzman, S. A. , Kramer, A. , Ball, A. S. , & Allinson, G. (2019). Elucidation of the microbial diversity in rivers in south‐West Victoria, Australia impacted by rural agricultural contamination (dairy farming). Ecotoxicology and Environmental Safety, 172, 356–363. 10.1016/j.ecoenv.2019.01.112 30731266

[fsn33721-bib-0002] Ahmed, W. , Bertsch, P. M. , Bivins, A. , Bibby, K. , Farkas, K. , Gathercole, A. , Haramoto, E. , Gyawali, P. , Korajkic, A. , McMinn, B. R. , Mueller, J. F. , Simpson, S. L. , Smith, W. J. M. , Symonds, E. M. , Thomas, K. V. , Verhagen, R. , & Kitajima, M. (2020). Comparison of virus concentration methods for the RT‐qPCR‐based recovery of murine hepatitis virus, a surrogate for SARS‐CoV‐2 from untreated wastewater. Science of the Total Environment, 739, 139960. 10.1016/j.scitotenv.2020.139960 32758945 PMC7273154

[fsn33721-bib-0003] Andani, A. , van Damme, P. , Bunge, E. M. , Salgado, F. , van Hoorn, R. C. , & Hoet, B. (2021). One or two doses of hepatitis a vaccine in universal vaccination programs in children in 2020: A systematic review. Vaccine, 40, 196–205. 10.1016/j.vaccine.2021.01.038 33526283

[fsn33721-bib-0004] Atmar, R. L. , Opekun, A. R. , Gilger, M. A. , Estes, M. K. , Crawford, S. E. , Neill, F. H. , Ramani, S., Hill,H., Ferrira, J. , & Graham, D. Y . (2013). Determination of the 50% human infectious dose for Norwalk virus. The Journal of Infectious Diseases, 209(7), 1016–1022. 10.1093/infdis/jit620 24253285 PMC3952671

[fsn33721-bib-0005] Bartsch, C. , Höper, D. , Mäde, D. , & Johne, R. (2018). Analysis of frozen strawberries involved in a large norovirus gastroenteritis outbreak using next generation sequencing and digital PCR. Food Microbiology, 76, 390–395. 10.1016/j.fm.2018.06.019 30166165

[fsn33721-bib-0006] Bertrand, I. , Schijven, J. F. , Sanchez, G. , Wyn‐Jones, P. , Ottoson, J. , Morin, T. , Muscillo, M. , Verani, M. , Nasser, A. , de Roda Husman, A. M. , Myrmel, M. , Sellwood, J. , Cook, N. , & Gantzer, C. (2012). The impact of temperature on the inactivation of enteric viruses in food and water: A review. Journal of Applied Microbiology, 112(6), 1059–1074. 10.1111/j.1365-2672.2012.05267.x 22380614

[fsn33721-bib-0007] Bosch, A. , Gkogka, E. , Le Guyader, F. S. , Loisy‐Hamon, F. , Lee, A. , van Lieshout, L. , Marthi, B. , Myrmel, M. , Sansom, A. , Schultz, A. C. , Winkler, A. , Zuber, S. , & Phister, T. (2018). Foodborne viruses: Detection, risk assessment, and control options in food processing. International Journal of Food Microbiology, 285, 110–128. 10.1016/j.ijfoodmicro.2018.06.001 30075465 PMC7132524

[fsn33721-bib-0008] Bouwknegt, M. , Verhaelen, K. , Rzeżutka, A. , Kozyra, I. , Maunula, L. , von Bonsdorff, C.‐H. , Vantarakis, A. , Kokkinos, P. , Petrovic, T. , Lazic, S. , Pavlik, I. , Vasickova, P. , Willems, K. A. , Havelaar, A. H. , Rutjes, S. A. , & de Roda Husman, A. M. (2015). Quantitative farm‐to‐fork risk assessment model for norovirus and hepatitis a virus in European leafy green vegetable and berry fruit supply chains. International Journal of Food Microbiology, 198, 50–58. 10.1016/j.ijfoodmicro.2014.12.013 25598201

[fsn33721-bib-0009] Brooks, H. A. , Gersberg, R. M. , & Dhar, A. K. (2005). Detection and quantification of hepatitis a virus in seawater via real‐time RT‐PCR. Journal of Virological Methods, 127(2), 109–118. 10.1016/j.jviromet.2005.03.017 15896854

[fsn33721-bib-0010] Butot, S. , Putallaz, T. , & Sánchez, G. (2008). Effects of sanitation, freezing and frozen storage on enteric viruses in berries and herbs. International Journal of Food Microbiology, 126(1), 30–35. 10.1016/j.ijfoodmicro.2008.04.033 18547667

[fsn33721-bib-0011] Cook, N. , Vickers‐Smith, L. , & D'Agostino, M. (2021). Detection of hepatitis a virus in strawberries implicated in an outbreak in the USA in 1997. Food and Environmental Virology, 13(3), 421–422. 10.1007/s12560-021-09480-2 34106432

[fsn33721-bib-0012] Danyluk, M. D. , & Schaffner, D. W. (2011). Quantitative assessment of the microbial risk of leafy greens from farm to consumption: Preliminary framework, data, and risk estimates. Journal of Food Protection, 74(5), 700–708. 10.4315/0362-028X.JFP-10-373 21549039

[fsn33721-bib-0013] de Matos Nascimento, A. , de Paula, V. R. , Dias, E. H. O. , da Costa Carneiro, J. , & Otenio, M. H. (2020). Quantitative microbial risk assessment of occupational and public risks associated with bioaerosols generated during the application of dairy cattle wastewater as biofertilizer. Science of the Total Environment, 745, 140711. 10.1016/j.scitotenv.2020.140711 32763641

[fsn33721-bib-0014] Flannery, J. , Keaveney, S. , Rajko‐Nenow, P. , Flaherty, V. , & Doré, W. (2012). Concentration of norovirus during wastewater treatment and its impact on oyster contamination. Applied and Environmental Microbiology, 78(9), 3400–3406. 10.1128/AEM.07569-11 22367079 PMC3346491

[fsn33721-bib-0015] Fraisse, A. , Coudray‐Meunier, C. , Martin‐Latil, S. , Hennechart‐Collette, C. , Delannoy, S. , Fach, P. , & Perelle, S. (2017). Digital RT‐PCR method for hepatitis a virus and norovirus quantification in soft berries. International Journal of Food Microbiology, 243, 36–45. 10.1016/j.ijfoodmicro.2016.11.022 27960104

[fsn33721-bib-0016] Gao, X. , Wang, Z. , Wang, Y. , Liu, Z. , Guan, X. , Ma, Y. , Zhou, H. , Jiang, Y., Cui, W., Wang, L., & Xu, Y. (2019). Surveillance of norovirus contamination in commercial fresh/frozen berries from Heilongjiang Province, China, using a TaqMan real‐time RT‐PCR assay. Food Microbiology, 82, 119–126. 10.1016/j.fm.2019.01.017 31027765

[fsn33721-bib-0017] Garcia Garrido, H. M. , Veurink, A. M. , Leeflang, M. , Spijker, R. , Goorhuis, A. , & Grobusch, M. P. (2019). Hepatitis a vaccine immunogenicity in patients using immunosuppressive drugs: A systematic review and meta‐analysis. Travel Medicine and Infectious Disease, 32, 101479. 10.1016/j.tmaid.2019.101479 31521804

[fsn33721-bib-0018] Gonzales‐Gustavson, E. , Rusiñol, M. , Medema, G. , Calvo, M. , & Girones, R. (2019). Quantitative risk assessment of norovirus and adenovirus for the use of reclaimed water to irrigate lettuce in Catalonia. Water Research, 153, 91–99. 10.1016/j.watres.2018.12.070 30703677

[fsn33721-bib-0019] Hasing, M. E. , & Pang, X. L. (2021). Norovirus: Molecular epidemiology, viral culture, immunity, and vaccines. Clinical Microbiology Newsletter, 43(5), 33–43. 10.1016/j.clinmicnews.2021.02.002

[fsn33721-bib-0020] Jahne, M. A. , Brinkman, N. E. , Keely, S. P. , Zimmerman, B. D. , Wheaton, E. A. , & Garland, J. L. (2020). Droplet digital PCR quantification of norovirus and adenovirus in decentralized wastewater and graywater collections: Implications for onsite reuse. Water Research, 169, 115213. 10.1016/j.watres.2019.115213 31671297 PMC7017454

[fsn33721-bib-0021] Kulsuptrakul, J. , Wang, R. , Meyers, N. L. , Ott, M. , & Puschnik, A. S. (2021). A genome‐wide CRISPR screen identifies UFMylation and TRAMP‐like complexes as host factors required for hepatitis a virus infection. Cell Reports, 34(11), 108859. 10.1016/j.celrep.2021.108859 33730579 PMC8893346

[fsn33721-bib-0022] León‐Félix, J. , Martínez‐Bustillos, R. A. , Báez‐Sañudo, M. , Peraza‐Garay, F. , & Chaidez, C. (2010). Norovirus contamination of bell pepper from handling during harvesting and packing. Food and Environmental Virology, 2(4), 211–217. 10.1007/s12560-010-9048-z

[fsn33721-bib-0023] Li, D. , Butot, S. , Zuber, S. , & Uyttendaele, M. (2018). Monitoring of foodborne viruses in berries and considerations on the use of RT‐PCR methods in surveillance. Food Control, 89, 235–240. 10.1016/j.foodcont.2018.02.024

[fsn33721-bib-0024] Li, Y. , Li, L. , Baochun, H. , Xuehua, L. , Junying, L. , & Jianjun, Z. (2005). Studies on variability of single fruit weight in strawberry. Hebei Agricultural Science and Technology, 9(1), 32–34.

[fsn33721-bib-0025] Miranda, R. C. , & Schaffner, D. W. (2018). Farm to fork quantitative microbial risk assessment for norovirus on frozen strawberries. Microbial Risk Analysis, 10, 44–53. 10.1016/j.mran.2018.06.002

[fsn33721-bib-0026] Mok, H.‐F. , Barker, S. F. , & Hamilton, A. J. (2014). A probabilistic quantitative microbial risk assessment model of norovirus disease burden from wastewater irrigation of vegetables in Shepparton, Australia. Water Research, 54, 347–362. 10.1016/j.watres.2014.01.060 24594660

[fsn33721-bib-0027] Mok, H. F. , & Hamilton, A. J. (2014). Exposure factors for wastewater‐irrigated Asian vegetables and a probabilistic rotavirus disease burden model for their consumption. Risk Analysis, 34(4), 602–613. 10.1111/risa.12178 24576153 PMC3984355

[fsn33721-bib-0028] Mukama, M. , Ambaw, A. , & Opara, U. L. (2020). Advances in design and performance evaluation of fresh fruit ventilated distribution packaging: A review. Food Packaging and Shelf Life, 24, 100472. 10.1016/j.fpsl.2020.100472

[fsn33721-bib-0029] Ortiz‐Solà, J. , Abadias, I. , Colàs‐Medà, P. , Anguera, M. , & Viñas, I. (2021). Inactivation of *Salmonella enterica*, *Listeria monocytogenes* and murine norovirus (MNV‐1) on fresh strawberries by conventional and water‐assisted ultraviolet light (UV‐C). Postharvest Biology and Technology, 174, 111447. 10.1016/j.postharvbio.2020.111447

[fsn33721-bib-0030] Ortiz‐Solà, J. , Abadias, M. , Colás‐Medà, P. , Sánchez, G. , Bobo, G. , & Viñas, I. (2020). Evaluation of a sanitizing washing step with different chemical disinfectants for the strawberry processing industry. International Journal of Food Microbiology, 334, 108810. 10.1016/j.ijfoodmicro.2020.108810 32805511

[fsn33721-bib-0031] Ortiz‐Solà, J. , Viñas, I. , Colás‐Medà, P. , Anguera, M. , & Abadias, M. (2020). Occurrence of selected viral and bacterial pathogens and microbiological quality of fresh and frozen strawberries sold in Spain. International Journal of Food Microbiology, 314, 108392. 10.1016/j.ijfoodmicro.2019.108392 31698282

[fsn33721-bib-0032] Ortúzar, J. E. , Dogan, O. B. , Sotomayor, G. , Jiménez, C. , Clarke, J. , Flores, R. A. , Gray, G. M., Rupnow, J. H., & Wang, B . (2020). Quantitative assessment of microbial quality and safety risk: A preliminary case study of strengthening raspberry supply system in Chile. Food Control, 113, 107166. 10.1016/j.foodcont.2020.107166

[fsn33721-bib-0033] Osuka, H. , Hall, A. J. , Wikswo, M. E. , Baker, J. M. , & Lopman, B. A. (2018). Temporal relationship between healthcare‐associated and nonhealthcare‐associated norovirus outbreaks and Google trends data in the United States. Infection Control and Hospital Epidemiology, 39(3), 355–358. 10.1017/ice.2017.322 29382406

[fsn33721-bib-0034] Predmore, A. , & Li, J. (2011). Enhanced removal of a human norovirus surrogate from fresh vegetables and fruits by a combination of surfactants and sanitizers. Applied and Environmental Microbiology, 77(14), 4829–4838. 10.1128/AEM.00174-11 21622782 PMC3147408

[fsn33721-bib-0035] Prez, V. E. , Martínez, L. C. , Victoria, M. , Giordano, M. O. , Masachessi, G. , Ré, V. E. , Pavan, J. V., Colina,R ., Barril, P. A., & Nates, S. V. (2018). Tracking enteric viruses in green vegetables from Central Argentina: Potential association with viral contamination of irrigation waters. Science of the Total Environment, 637‐638, 665–671. 10.1016/j.scitotenv.2018.05.044 29758423

[fsn33721-bib-0036] Regulation, S. A. F. M . (2015). Local food safety standard: Norovirus detection in food. State Administration for Market Regulation.

[fsn33721-bib-0037] Regulation, S. A. F. M . (2016). National food safety standard: Microbiological examination of food norovirus examination. State Administration for Market Regulation.

[fsn33721-bib-0038] Rodrigues, C. , da Silva, A. L. B. R. , & Dunn, L. L. (2020). Factors impacting the prevalence of foodborne pathogens in agricultural water sources in the southeastern United States. Water, 12(1), 51. 10.3390/w12010051

[fsn33721-bib-0039] Sun, C. , Chen, J. , Li, H. , Fang, L. , Wu, S. , Jayavanth, P. , Tang, S., Sanchez, G., & Wu, X . (2021). One‐step duplex RT‐droplet digital PCR assay for the detection of norovirus GI and GII in lettuce and strawberry. Food Microbiology, 94, 103653. 10.1016/j.fm.2020.103653 33279078

[fsn33721-bib-0040] Sun, Q. , Cao, M. , Zhang, X. , Wang, M. , Ma, Y. , & Wang, J. (2021). A simple and low‐cost paper‐based colorimetric method for detecting and distinguishing the GII.4 and GII.17 genotypes of norovirus. Talanta, 225, 121978. 10.1016/j.talanta.2020.121978 33592726

[fsn33721-bib-0041] Takahashi, M. , Okakura, Y. , Takahashi, H. , Imamura, M. , Takeuchi, A. , Shidara, H. , Kuda, T., & Kimura, B . (2018). Heat‐denatured lysozyme could be a novel disinfectant for reducing hepatitis a virus and murine norovirus on berry fruit. International Journal of Food Microbiology, 266, 104–108. 10.1016/j.ijfoodmicro.2017.11.017 29202339

[fsn33721-bib-0042] Verhaelen, K. , Bouwknegt, M. , Carratalà, A. , Lodder‐Verschoor, F. , Diez‐Valcarce, M. , Rodríguez‐Lázaro, D. , de Roda Husman, A. M., & Rutjes, S. A. (2013). Virus transfer proportions between gloved fingertips, soft berries, and lettuce, and associated health risks. International Journal of Food Microbiology, 166(3), 419–425. 10.1016/j.ijfoodmicro.2013.07.025 24029026

[fsn33721-bib-0043] Wang, L. , Ting, J. S. L. , & Ip, W. H. (2013). Design of supply‐chain pedigree interactive dynamic explore (SPIDER) for food safety and implementation of Hazard analysis and critical control points (HACCPs). Computers and Electronics in Agriculture, 90, 14–23. 10.1016/j.compag.2012.10.004

[fsn33721-bib-0044] Wu, C.‐Y. , Chi, H. , Liu, C.‐C. , Huang, Y.‐C. , Huang, Y.‐C. , Lin, H.‐C. , Ho, Y.‐H., Huang, L‐.M., Huang, C‐.Y., Shih, S‐.M., Wu, F‐.T., Mu, J‐.J., & Hsiung, C. A. (2020). Clinical characteristics and risk factors for children with norovirus gastroenteritis in Taiwan. Journal of Microbiology, Immunology, and Infection, 54, 909–917. 10.1016/j.jmii.2020.07.013 32943327

[fsn33721-bib-0045] Yao, B. , Luo, Z. , Xiong, W. , Song, B. , Zeng, Z. , & Zhou, Y. (2020). Disinfection techniques of human norovirus in municipal wastewater: Challenges and future perspectives. Current Opinion in Environmental Science & Health, 17, 29–34. 10.1016/j.coesh.2020.08.003

[fsn33721-bib-0046] Yeargin, T. A. , Fraser, A. M. , & Gibson, K. E. (2021). Characterization of risk management practices among strawberry growers in the southeastern United States and the factors associated with implementation. Food Control, 122, 107758. 10.1016/j.foodcont.2020.107758

